# Toxicity screening of bisphenol A replacement compounds: cytotoxicity and mRNA expression in LMH 3D spheroids

**DOI:** 10.1007/s11356-022-18812-z

**Published:** 2022-02-09

**Authors:** Tasnia Sharin, Doug Crump, Jason M. O’Brien

**Affiliations:** 1 Environment and Climate Change Canada, National Wildlife Research Centre, Ottawa, ON K1S 5B6 Canada; 2 Department of Biology, University of Ottawa, Ottawa, ON K1N 6N5 Canada

**Keywords:** Bisphenol A, Hepatocytes, Gene expression, Cytotoxicity, 3D spheroids

## Abstract

Previously, we showed that the chicken LMH cell line cultured as 3D spheroids may be a suitable animal free alternative to primary chicken embryonic hepatocytes (CEH) for avian in vitro chemical screening. In this study, cytotoxicity and mRNA expression were determined in LMH 3D spheroids following exposure to bisphenol A (BPA), five BPA replacement compounds (BPF, TGSH, DD-70, BPAF, BPSIP), and 17β estradiol (E2). Results were compared to an earlier study that evaluated the same endpoints for these chemicals in CEH. BPA and the replacement compounds had LC50 values ranging from 16.6 to 81.8 μM; DD-70 and BPAF were the most cytotoxic replacements (LC50 = 17.23 ± 4.51 and 16.6 ± 4.78 μM). TGSH and DD-70 modulated the greatest number of genes, although fewer than observed in CEH. Based on the expression of apovitellenin and vitellogenin, BPAF was the most estrogenic compound followed by BPF, BPSIP, and BPA. More estrogen-responsive genes were modulated in LMH spheroids compared to CEH. Concentration-dependent gene expression revealed that DD-70 and BPAF altered genes related to lipid and bile acid regulation. Overall, cytotoxicity and clustering of replacements based on gene expression profiles were similar between LMH spheroids and CEH. In addition to generating novel gene expression data for five BPA replacement compounds in an in vitro avian model, this research demonstrates that LMH spheroids may represent a useful animal free alternative for avian toxicity testing.

**Supplementary Information:**

The online version contains supplementary material available at 10.1007/s11356-022-18812-z.

## Introduction

In recent years, toxicity testing has shifted focus towards mechanistic approaches to characterize the toxicity of the large number of chemicals in the environment (Krewski et al. 2009). Primary embryonic hepatocytes are frequently used for avian toxicity testing (Mundy et al. 2019; Porter et al. 2014). Primary hepatocytes have similar gene expression and enzyme activity compared to in vivo conditions; however, a loss of biochemical functions occurs shortly after isolation (Fraczek et al. 2013). Advancements in 3D cell culture techniques, such as spheroids, which resemble 3D organization of cells in vivo, have become more common in toxicity testing applications (Hellwig et al. 2018; Breslin and O'Driscoll 2013). Immortalized hepatic cell lines are an animal free alternative to primary hepatocytes and when cultured as spheroids have improved gene expression and metabolic/biochemical activities and are thus better predictors of toxicity than 2D monolayer cultures (Sharin et al. 2020; Ramaiaghari et al. 2014).

Bisphenol A (BPA) replacement compounds are a group of chemicals for which toxicity data are limited. BPA is an industrial chemical used in the production of polycarbonate plastics and epoxy resin in a wide range of consumer products. BPA can bind to endocrine receptors and interfere with other biological pathways, which led to a ban on use in applications including infant products, food packaging, and thermal paper (Siracusa et al. 2018; ECHA 2020). Such restrictions have led to the production of “BPA-free” products, many of which contain structural analogs of BPA. The production and use of BPA replacement compounds are increasing globally, and they have been detected in various environmental samples (Chen et al. 2016), yet their toxic effects remain largely unknown. Thus, there is a strong demand to rapidly generate ecotoxicological information for this group of priority compounds.

There is increasing pressure to reduce the number of animals used for toxicity testing/screening, and previous studies from our laboratory have used primary chicken embryonic hepatocytes (CEH) to screen a variety of chemicals (Page-Lariviere et al. 2018; Porter et al. 2014), the preparation of which requires animals. Here, we apply an in vitro transcriptomic approach to generate toxicity data for five BPA replacement compounds in an immortalized avian hepatic cell line. The chicken leghorn male hepatoma (LMH) cell line, especially when cultured as 3D spheroids, is a potential alternative to CEH for avian toxicity testing due to enhanced metabolic activity and gene expression (Sharin et al. 2020). The five BPA replacement compounds chosen for this study are as follows: bisphenol F (BPF), 4,4′-sulfonylbis(2-allylphenol) (TGSH/TGSA), phenol, 4,4′-[methylenebis(oxy-2,1-ethanediylthio)] bis- (DD-70), bisphenol AF (BPAF), and 4-hydroxyphenyl 4-isopropoxyphenyl sulfone (BPSIP/D-8). BPF was detected in surface water in Asia (~ 1000 ng/L; Chen et al. 2016) and in wildlife, including the liver (3.01 ng/g wet weight) of the white-tailed eagle (*Haliaeetus albicilla*) (Gonzalez-Rubio et al. 2020). BPAF has been detected at similar concentrations as BPA (0.9–246 ng/L) in river water in China (Wang et al. 2017). There is no information available on the environmental occurrence of TGSH, DD-70, or BPSIP. TGSH was more teratogenic than BPA in zebrafish embryos (Bjornsdotter et al. 2017) and had similar lethality as BPA in chicken embryos (Crump et al. 2018). DD-70 was found to be non-estrogenic in a reporter gene assay (Keminer et al. 2020). Several studies have found BPAF to be more cytotoxic and estrogenic than BPA in several cell lines and species (Mu et al. 2018; Song et al. 2014; Kitamura et al. 2005). BPSIP upregulated mRNA expression of estrogen receptors (Lee et al. 2018) and caused similar developmental abnormalities as BPA in zebrafish embryos (Bjornsdotter et al. 2017). Similar to environmental occurrence, there are limited data on the potential toxicity of TGSH, DD-70 and BPSIP.

The specific aims of this study were to (1) generate toxicity data for five BPA replacement compounds in LMH 3D spheroids, including cytotoxicity and perturbations in gene expression, and (2) evaluate whether the immortalized cell line, LMH, when cultured as 3D spheroids, is an effective in vitro model for avian hepatic toxicity, and a viable animal free alternative to CEH for chemical screening based on comparisons with recently reported results for the same five chemicals in CEH (Sharin et al. 2021a).

## Materials and methods

### Reagents

BPF (98%), BPA (97%), and 17β estradiol (E2, 98%) were purchased from Sigma-Aldrich; TGSH (96%), BPAF (98%), and BPSIP (98%) from Santa Cruz Biotechnology; and DD-70 (97%) from Toronto Research Chemicals (Table 1). The certificate of analysis provided by the supplier of the chemicals confirmed that the chemicals met quality control specifications. Stock solutions of the chemicals were prepared by dissolution in dimethyl sulfoxide (DMSO; Sigma-Aldrich) to a final concentration of 1 mM, except for E2 (200 μM). Dosing solutions were prepared by serial dilution from stock solutions to attain nominal concentrations of 0.1 to 100 μM. All cell culture reagents were purchased from Sigma-Aldrich unless stated otherwise.

**Table 1:**
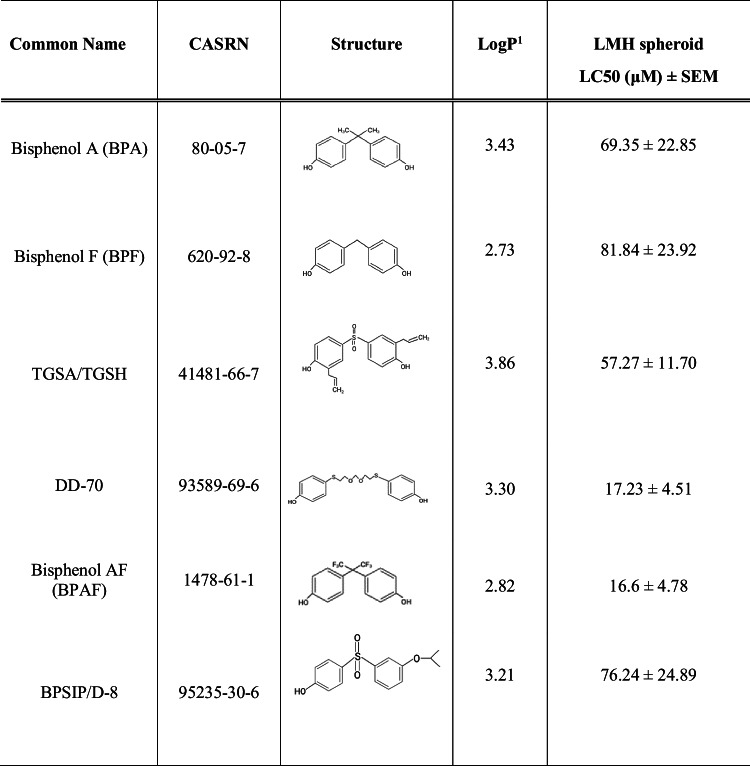
Common name, CASRN, structure, and logP of bisphenol A (BPA) and five BPA replacement compounds and their corresponding LC50 values (μM) ± SEM in LMH 3D spheroids

^1^LogP values from ACD lab on Chemspider.

### Cell culture, dosing, and viability

LMH cells were cultured, maintained, and characterized as previously described (Sharin et al. 2020). For 3D spheroids, cells were seeded at 10 000 cells/well in 250μl medium/well in ultra-low attachment (ULA) 96 well microplates (Corning) and grown for 4 days. Medium was changed every day during the 4 day period by removing 200μL and adding 200μL of fresh medium to each well. The cells formed compact spheroids with smooth edges by 48h. On day 5, spheroids without visible necrotic cores (~ 200μm in diameter) were treated with the DMSO solvent control and 0.1 to 100μM BPA or the five replacement compounds (0.5% v/v in 250μL medium) for cell viability evaluation (*n* = 3/treatment group). For gene expression analysis, spheroids were exposed to 30 μM BPF, TGSH, BPSIP, and BPA and 10 μM DD-70, BPAF, and E2 (*n* = 3/treatment group). E2 was used as a positive control for gene expression analysis. Non-cytotoxic concentrations of BPA and replacement compounds were chosen, based on the LC50 values we determined, for gene expression analysis. Finally, LMH spheroids were treated with 0.01, 0.1, and 1μM of DD-70 and BPAF for concentration-dependent gene expression evaluation. Spheroids were exposed for 24h and immediately assayed for cell viability determination or stored at -80°C until subsequent gene expression analysis.

Cell viability was determined using the CellTiter Glo 3D assay (catalog no. G9682, Promega) following the manufacturer’s instructions. Briefly, after the 24h exposure period, 100μL of assay reagent was added to each well containing spheroids in 100μL medium and placed on an orbital shaker for lysis. The contents of each well were transferred to a flat clear bottom white 96 well microplate (Nunc) and incubated for 10 min in the dark at room temperature, and luminescence was read using the Luminoskan Ascent Luminometer (ThermoFisher).

### RNA isolation and cDNA synthesis

Total RNA was extracted from spheroids using the PureLink RNA Micro Scale kit (catalog no. 12183016, ThermoFisher) according to the manufacturer’s protocol. Two spheroids per replicate (*n* = 3) were pooled to ensure sufficient RNA yield. RNA concentration was quantified using a NanoDrop 2000 (ThermoFisher), and 250ng was reverse transcribed with the QuantiTect Reverse Transcription kit (catalog no. 205314, Qiagen) according to the manufacturer’s instructions.

### PCR arrays

Gene expression was determined using three customized chicken PCR arrays: (1) ToxChip, (2) AestroChip, and (3) ComboChip. The ToxChip array (catalog no. CLAG22572, Qiagen, Table S1) comprises 43 target genes covering eight biologically relevant pathways, 2 reference genes (EEF1A1 and RPL4), and 3 quality controls (genomic DNA contamination control, reverse transcription control, and a positive PCR control). The AestroChip array consists of 9 estrogen-responsive genes, 2 reference genes (EEF1A1 and RPL4), and a no template control (NTC) (Table S2). Concentration-dependent gene expression of DD-70 and BPAF were determined using the ComboChip array, which consists of 4 genes from the AestroChip and 6 genes from the ToxChip, 2 reference genes (EFF1A1 and RPL4), and a NTC (Table S3). The RT^2^ qPCR primer assays for the AestroChip and ComboChip arrays were purchased from Qiagen, and thus the sequences are proprietary. Real-time RT-PCR reactions were prepared with the RT^2^ SYBR Green qPCR Master Mix kit (Qiagen). The thermal profile was 95°C for 10 min followed by 40 cycles of 95°C for 15s and 60°C for 1 min and a final extension at 95°C for 1 min. Real‐time RT‐PCR was performed using a Stratagene Mx3005 instrument (Agilent Technologies) and CFX96 Touch Real-Time PCR Detection System (Bio-Rad Laboratories).

### Data analysis

To calculate the lethal median concentration (LC50), concentrations were log transformed, and luminescence values were normalized and fit to a nonlinear regression curve (log (agonist) vs. response) using least squares (ordinary) fit on GraphPad Prism Ver. 6.07 (Fig. S1). Two-way ANOVA and post hoc Sidak comparison of LC50 values in LMH spheroids and CEH (Sharin et al. 2021a) were performed using GraphPad Prism Ver. 6.07.

The reverse transcription, genomic DNA, and positive PCR controls met the appropriate quality control/assurance criteria for the ToxChip, and there was no amplification in the NTC for the AestroChip or ComboChip. All PCR array cycle threshold (Ct) values were normalized to the housekeeping gene RPL4 using the 2^−ΔCt^ method (Schmittgen and Livak 2008). EEF1A1 was omitted as a reference gene due to variabilities in expression in the BPA samples on the AestroChip array. Genes on the ToxChip array with missing Ct values or Ct values above 35 in more than 25% of all samples were removed from analysis. Fold change was determined relative to the DMSO control group. The fold change data were log2-transformed before ANOVA analysis to account for unequal variance. The resulting *p* values from pairwise comparisons between control and treated groups for all genes were adjusted using the Benjamini–Hochberg method with the false discovery rate (FDR) fixed at 5%. Principal component analysis (PCA) was performed on log2-transformed fold-change data using the prcomp function in R with the default settings (R Core Team 2013) and visualized using the autoplot function in the ggplot2 package (Wickham 2016).

## Results and discussion

BPA replacements are of emerging concern due to their potential to perturb endocrine systems and other pathways. Environmental contamination of these compounds is expected to increase as BPA is being phased out, yet there is limited information on their toxicological properties (Rosenmai et al. 2014). Therefore, a rapid approach is needed to identify any potential toxicological concerns these replacement compounds may present. Ideally, any approach would also be as ethical as possible in terms of animal utilization. In the present study, we utilized the immortalized chicken hepatic cell line, LMH, cultured as spheroids, to screen five BPA replacement compounds. Results using this animal free in vitro screening approach were compared to those previously reported for the same five replacement compounds in another in vitro model that required animal use, CEH (Sharin et al. 2021a).

The LC50 values of two of the replacement compounds, DD-70 and BPAF, were 17.23 ± 4.51 and 16.6 ± 4.78µM (Table 1, Fig. S2), which were similar to LC50 values in CEH (Sharin et al. 2021a). Both DD-70 and BPAF were more cytotoxic than BPA in both LMH spheroids and CEH. For BPA, the LC50 value was 69.35 ± 22.85µM, which was similar to the LC50 determined in CEH, 61.7 ± 43.1µM (Ma et al. 2015). TGSH had a LC50 value of 57.27 ± 11.70µM, and the two other replacement compounds, BPSIP and BPF, had LC50 values of 76.24 ± 24.89 and 81.84 ± 23.92µM, respectively. BPF was more cytotoxic in LMH spheroids than in CEH (LC50 between 100 and 300µM). The rank order of the chemicals evaluated in the present study based on LC50 in LMH spheroids was BPAF ~ DD-70 > TGSH ~ BPA ~ BPSIP ~ BPF. BPAF and BPF were one of the most and least cytotoxic, respectively, among several BPA replacement compounds screened in various cell lines (Liu et al. 2020; Russo et al. 2018; Goldinger et al. 2015). No other cell viability data exist for TSGH or DD-70. BPSIP was found to have similar cytotoxicity as BPA in H295R adrenocortical cell line (Goldinger et al. 2015). The LC50 values of BPA (69.35µM) and BPSIP (76.24µM) were also similar in LMH spheroids in the present study.

The log octanol–water partition coefficient (logP) values of BPA and the replacement compounds are between ~ 2.7 and 3.9 (Table 1). Compounds with logP values ≤ 5 are hydrophilic, do not permeate the cell membrane easily, and, therefore, tend to have low cytotoxicity (Yang and Hinner 2015). In a previous study, bisphenol replacements with logP ≥ 5 had increased cytotoxicity (Crump et al. 2021). DD-70 and BPAF have low logP values, 3.30 and 2.82, but were the most cytotoxic among the compounds evaluated. Structurally, the scaffold between the phenol rings of DD-70 is the most varied among the compounds evaluated. DD-70 has two ethers and two sulfides between the phenol rings (Table 1), which could alter chemical reactivity and cytotoxicity. BPAF has two trifluoromethyl groups attached to the carbon bridging the two phenols (Table 1) that are highly electronegative, hydrophobic, and reactive (Filler and Saha 2009), which could account for the high cytotoxicity.

**Fig. 1:**
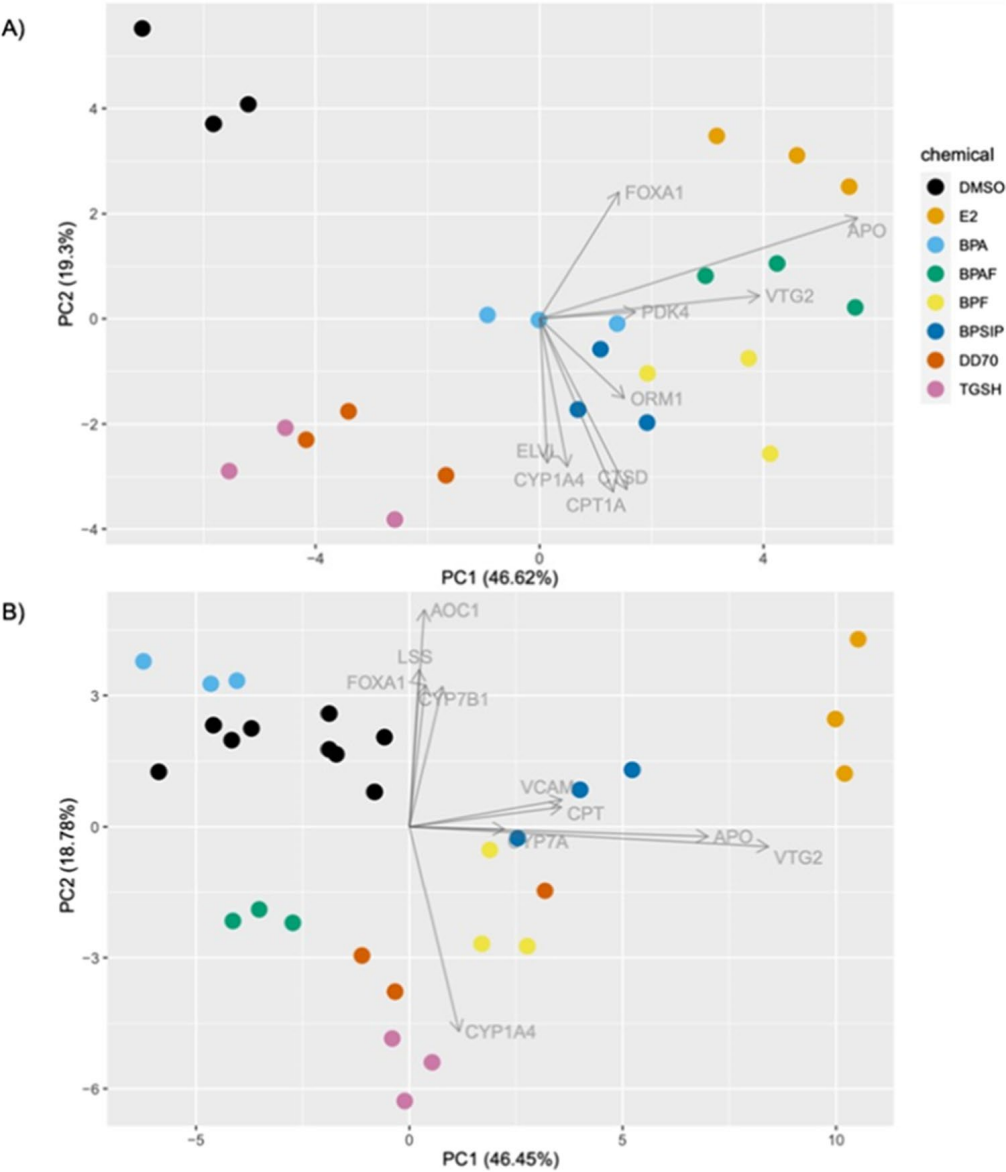
Principal component analysis of significant genes on the ToxChip and AestroChip arrays in **A**) LMH 3D spheroids and **B**) CEH following exposure to BPA, BPA replacement compounds (BPF, TGSH, DD-70, BPAF, and BPSIP), and 17β estradiol (E2). Data are colored by chemical. Gene loadings for the most responsive transcripts are depicted by arrows on the graph. CEH data are from Sharin et al. (2021a)

The expression of ToxChip and AestroChip genes in LMH spheroids were combined and reduced to two principal components using PCA (Fig. 1A). The first principal component (PC1) accounted for 46.6% of the variability and PC2 explained 19.3% of the variability among samples. The key contributors to PC1 were the AestroChip genes, apovitellenin (APO), and vitellogenin II (VTG2), while PC2 clustering was based on ToxChip and AestroChip genes including cytochrome P450 1A4 (CYP1A4) and carnitine palmitoyltransferase 1A (CPT1A). Similarly, PCA of combined ToxChip and AestroChip gene expression in CEH showed that PC1 was mainly based on VTG2 and APO, whereas ToxChip genes explained PC2 variability (Fig. 1B) (Sharin et al. 2021a). In LHM spheroids, BPAF and BPF clustered closely with E2 along PC1, suggesting that that these two compounds may have estrogenic properties. TGSH and DD-70 separated furthest from the DMSO controls along PC2 in LMH spheroids, suggesting non-estrogenic toxicity. Compared to LMH, BPAF did not cluster with E2, but rather separated along PC2 with TGSH and DD-70 in CEH. BPF clustered towards E2 in CEH, but not as strongly as in LMH. BPSIP clustered in a similar manner in LMH spheroids and CEH, somewhere in between DMSO controls and E2 along PC1, indicating modest estrogenicity in both models. BPA was more transcriptionally active in LMH spheroids compared to CEH but was still closest to the DMSO control. This is an important consistency because the more transcriptionally responsive chemicals are those that could potentially replace BPA in various applications (Crump et al. 2021).

The modulation of AestroChip genes by BPA and the replacement compounds was greater in LMH spheroids than in CEH. The rank order of dysregulation of APO and VTG2 expression in LMH spheroids was as follows: E2 (6.98 and 6.68-log2), BPAF (6.36 and 4.41-log2), BPF (5.76 and 4.18-log2), BPSIP (3.21 and 3.74-log2), BPA (2.29 and 3.76-log2), and DD-70 (no change and 2.56-log2) (Table S4). In CEH, VTG2 was down regulated by BPAF (-2.9 log2) and BPA (-2.8 log2), while none of the other replacement compounds altered VTG2 expression (Sharin et al. 2021a). For the preparation of CEH cultures, livers from females and males were pooled, and the evaluation of VTG2 expression in this mixed sex pool could help explain the discrepancy in VTG2 expression between CEH and LMH spheroids. The increased sensitivity of the LMH spheroids to estrogen-responsive genes compared to CEH is likely due to fact that the LMH cell line was established from a male liver. Males are typically more vulnerable to the adverse effects of xenoestrogens, and the inductions of estrogen responsive genes (e.g., VTG) are more evident since the basal expressions of these genes are low in adult males (Matozzo et al. 2008).

Several BPA replacement compounds had gene expression profiles in LMH that were more similar to E2 compared to BPA. Specifically, BPAF, BPF, and BPSIP all had stronger VTG2 and APO induction than BPA. VTG2, a marker of endocrine disruption, is synthesized by the liver in response to estrogen (Woods and Kumar 2011). APO is an egg yolk protein and lipoprotein lipase inhibitor involved in preventing the breakdown of very low-density lipoproteins (Bourin et al. 2012). VTG2 and APO are established markers of xenoestrogen exposure in oviparous species (Li et al. 2014; Heppell et al. 1995) and were the most responsive AestroChip genes for the PCA analysis (Fig. 1A). Similar to the current study, BPAF was more estrogenic than BPF and BPA in zebrafish embryo-larvae (Moreman et al. 2017) and increased VTG expression in zebrafish liver (Yang et al. 2014). Other studies have found BPAF was more estrogenic than BPA in several cell lines (Russo et al. 2018; Chen et al. 2016; Fic et al. 2014). BPF exposure resulted in the upregulation of vitellogenin (concordant with the findings in LMH spheroids in the present study) and aromatase (CYP19A1B) via estrogen receptor binding in zebrafish (Le Fol et al. 2017; Cano-Nicolau et al. 2016). TGSH had no significant estrogen receptor binding in reporter gene assays and did not change VTG2 expression in chickens (Bjornsdotter et al. 2017; Crump et al. 2018). Similarly, DD-70 did not bind to the estrogen receptor (Keminer et al. 2020). BPSIP was found to alter estradiol concentrations in the human H295R cell line, although it was less estrogenic than BPA (Goldinger et al. 2015; Bjornsdotter et al. 2017). The overall ranking of the compounds on the AestroChip in terms of VTG2 and APO modulation was as follows: E2 > BPAF > BPF > BPSIP > BPA > DD-70 > TGSH (Table S4).

In general, all of the compounds led to fewer dysregulated genes on the ToxChip array compared to the AestroChip array in LMH spheroids suggesting that the compounds have minor effects on non-estrogenic pathways. TGSH, DD-70, and BPSIP altered the most genes on the ToxChip array, and the expression of CYP1A4 was upregulated by all five replacement compounds, similar to the results in CEH (Sharin et al. 2021a). In the human hepatic cell line, HepG2, BPF, and BPAF upregulated the expression of CYP1A1 (Hercog et al. 2019). The transcription of CYP1A4 is regulated by AhR, and therefore, the five replacement compounds are possible AhR agonists (Monostory et al. 2009). The limited response of ToxChip genes in LMH spheroids compared to CEH could be related to developmental stage. That is, CEH are cultured from embryonic livers, and developing organs are more vulnerable to chemical toxicity compared to adults (Barton et al. 2005); the LMH cell line was established from an adult. The toxicity of the compounds on the ToxChip in LMH cells cultured as 2D monolayers needs to be investigated in order to determine if the discrepancy in chemical response could be due to different culture conditions. The rank order of the replacement compounds based on number of genes altered on the ToxChip was as follows: DD-70 > TGSH = BPSIP > BPF > BPAF = BPA > E2. The fold changes of genes on the ToxChip array following exposure to all of the compounds are in Table S5. 

**Fig. 2:**
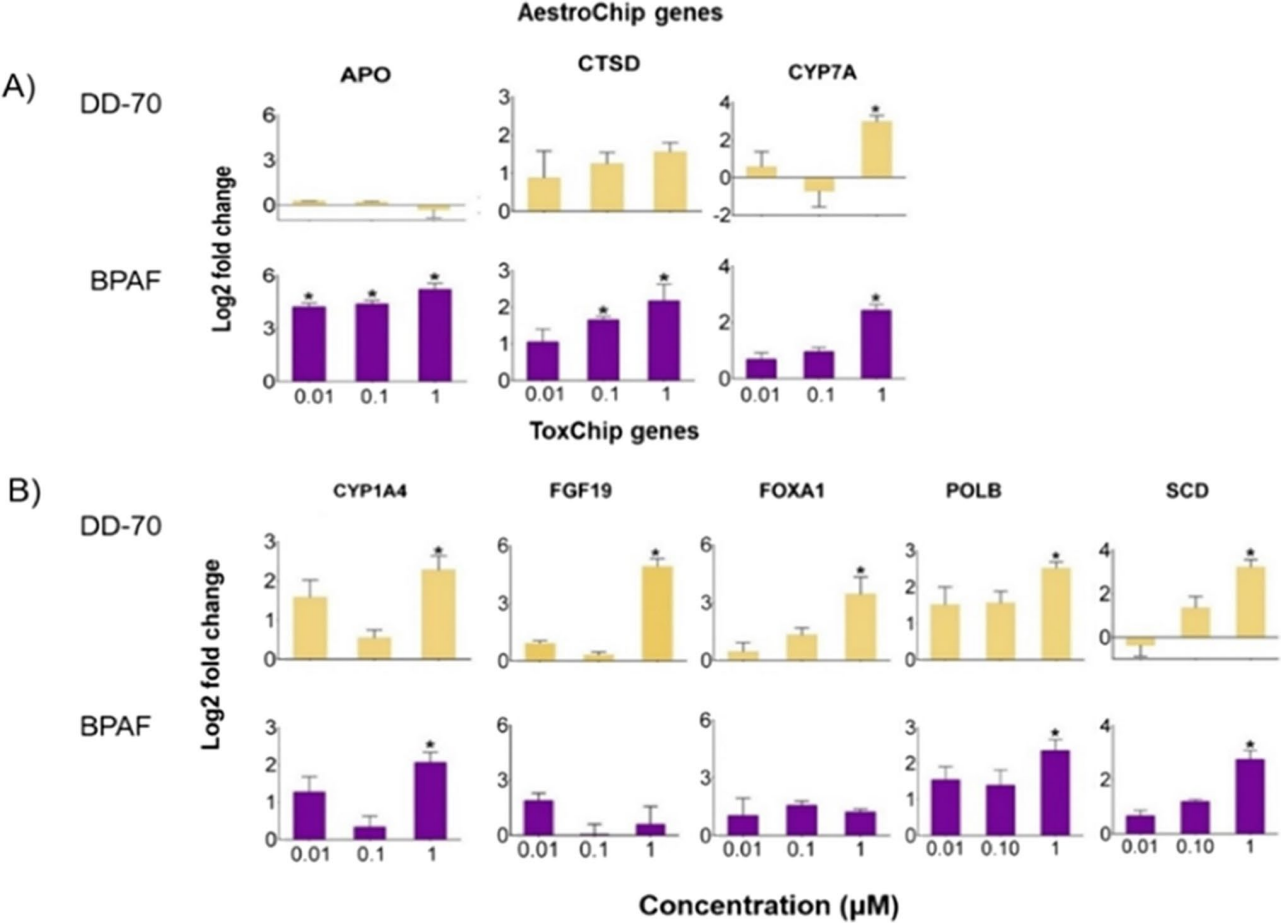
Concentration-dependent fold change (log2) in significant expression of selected **A** AestroChip and **B** ToxChip genes following exposure to DD-70 or BPAF for 24 h in LMH spheroids. The asterisk denotes difference compared to DMSO treated spheroids

DD-70 and BPAF were chosen for further concentration-dependent gene expression analysis using the ComboChip array (Fig. 2; Table S3). DD-70 was selected because it was one of the most cytotoxic replacements, yet appeared to have a non-estrogenic mode of action, based on our PCA results. Moreover, there is very little toxicity data available for DD-70. BPAF was selected due to its high cytotoxicity and because it was more estrogenic than BPA based on our LMH PCA results and other studies (Russo et al. 2018; Chen et al. 2016; Fic et al. 2014). The genes selected for the array were those that were most dysregulated from both arrays in CEH. Both DD-70 and BPAF upregulated the expression of CYP1A4, cholesterol 7α-hydroxylase (CYP7A1), stearoyl-CoA desaturase (SCD) and polymerase β (POLB) (Fig. 2; Table S6). Both compounds upregulated CYP1A4 at an administered concentration of 1 µM, which was sustained at 10 µM on the ToxChip array. CYP7A is the rate-limiting enzyme in bile acid synthesis (Jelinek et al. 1990), and SCD is involved in the synthesis of monounsaturated fatty acids (Piccinin et al. 2019). Another replacement compound, BPS, upregulated the hepatic expression of CYP7B1, which is involved in the alternative pathway of bile acid synthesis in chickens (Crump et al. 2016). In previous studies, BPAF upregulated the expression of SCD and other lipid metabolism genes in HepG2 and 3T3-L1cells (Liu et al. 2020; Skledar et al. 2019). POLB is involved in base excision repair by incorporating the correct base at the site of single stranded breaks (Martin et al. 2010). BPAF (30 µM) induced DNA double strand breaks; upregulated the expression of cell cycle genes, CDKN1A and GADD45, in HepG2 cells (Hercog et al. 2019); and induced single strand breaks at 3 nM in peripheral blood mononuclear cells (Mokra et al. 2017). DD-70 did not change the expression of the estrogen-responsive genes, APO and VTG2, at the doses included on the ComboChip array corroborating results in CEH. Two genes were upregulated solely by DD-70 following evaluation with the ComboChip: fibroblast growth factor 19 (FGF19) and forkhead box A1 (FOXA1) (Fig. 2B; Table S6). These gene targets are involved in maintaining bile acid homeostasis (Schumacher and Guo 2016) and bile duct development (Bernardo and Keri 2012). Such dysregulation is consistent with another study that found an increase in FGF19 expression in livers of chicken embryos exposed to DD-70 via egg injection (Sharin et al. 2021b). Overall, DD-70 and BPAF exposure may lead to disruption of bile acid and lipid regulation; however, further studies are warranted to evaluate this.

Specific to BPAF, we observed concentration-dependent increases in APO and cathepsin D (CTSD) expression starting at 0.01 and 0.1µM, respectively, while no change in VTG2 expression was observed (Fig. 2A). On the AestroChip array, 10µM BPAF upregulated the expression of APO, CTSD and VTG2; therefore, in the concentration-dependent study, the threshold concentration for induction of VTG2 by BPAF was not achieved. BPAF strongly binds to both estrogen receptors α (ERα) and β (ERβ), with binding potencies 20 and 48 times greater than BPA (Matsushima et al. 2010). CTSD is a protease that cleaves proteins to activate and deactivate enzymes (Benes et al. 2008), and its expression is mediated by estrogen (Bretschneider et al. 2008). Similar to LMH spheroids, BPAF upregulated CTSD expression in a concentration-dependent (0.01 to 10µM) manner in the MCF-7 cell line (Li et al. 2014). Overall, BPAF induced estrogen responsive genes in LMH spheroids, while DD-70 was not estrogenic at the lower concentrations evaluated.

The use of LMH spheroids permitted a concentration-dependent analysis of DD-70 and BPAF effects, an approach that would have been more logistically challenging with CEH due to the time-consuming nature of the culture and limited availability of primary hepatocytes for a single study. On the ToxChip, DD-70 and BPAF (10 µM) altered 3 and 0 genes related to bile acid and lipid homeostasis, respectively. In contrast, 7 and 3 genes from the same pathways were modulated by DD-70 and BPAF (10 µM) in CEH. At lower doses, the expressions of genes associated with this pathway on the ComboChip array were altered, indicating that bile and lipid genes are modulated by DD-70 and especially BPAF. The results from the ComboChip array emphasize the importance of concentration-dependent analysis in terms of gene expression as some genes were modulated at low doses, while other genes like VTG2 were altered at a higher dose. This dynamic gene expression response, which is reliant on dose, highlights the importance of future studies that incorporate concentration-dependent gene expression analysis when trying to determine the mechanism of action of chemicals and gene expression points of departure.

In summary, the ranking of the five replacement compounds in terms of cytotoxicity was similar in LMH spheroids and CEH. In terms of gene expression, the replacement compounds upregulated CYP1A expression in both models. TGSH and DD-70 were non-estrogenic compared to the other replacements, while BPAF was the most estrogenic replacement based on the gene expression endpoints measured in LMH spheroids. Gene alterations on the AestroChip array were more pronounced in LMH spheroids compared to CEH suggesting that LMH may be a more sensitive model for detecting estrogenic activity. The concentration-dependent analysis revealed that DD-70 and BPAF modulated the expression of genes related to bile acid and lipid metabolism pathways. Overall, LMH spheroids represent a useful, animal free model for screening xenoestrogens and a suitable alternative to CEH for avian toxicity testing.

## Supplementary Information

Below is the link to the electronic supplementary material.

Supplementary file1 (486 KB)

## Data Availability

The datasets used and/or analyzed during the current study are available from the corresponding author (jason.obrien@ec.gc.ca) on reasonable request.
